# Stamens enclosed by petals in *Berchemia* (Rhamnaceae): a unique mechanism for pollen presentation

**DOI:** 10.3389/fpls.2025.1525022

**Published:** 2025-01-29

**Authors:** Fang Ma, Qian Zhao, Xing Tian, Yao-lei Fu, Wen-zhe Liu

**Affiliations:** ^1^ School of Life Science, Northwest University, Xi’an, China; ^2^ Institute of Ethnic Preparatory Education, Ningxia University, Yinchuan, China

**Keywords:** *Berchemia*, secondary pollen presentation, petal-stamen complex, sexual interference, delayed selfing, reproduction assurance

## Abstract

**Introduction:**

Pollen is usually presented by the anthers after maturity. However, in some plants, pollen is presented to pollinators on other floral structures (other than the anthers), or via particular expulsion mechanisms, resulting in secondary pollen presentation. The unusual petal morphology in *Berchemia* mediates pollen presentation, characterised by a combination of secondary pollen presentation and primary pollen presentation. However, the function, the role in reproduction, and the evolutionary significance of the unusual petals remain unclear.

**Methods:**

In this study, we took *Berchemia flavescens* and *Berchemia polyphylla* var. *leioclada* as examples, and used field observations, semi-thin sections technology, scanning electron microscopy, and pollination ecology detection methods to explore the unique pollen presentation strategies, petal functions, and reproductive strategies in *Berchemia*.

**Results:**

This is a unique pollen presentation process mediated by petals. In the advanced bud stage, petals curl inward, enclosing the stamens. Following anther dehiscence, pollen is released into the petal tube, where the filament and cone-shaped anthers act as pistons, extruding pollen or pollen clumps through gaps at the petal tube apex (secondary pollen presentation). Subsequently, as the anthers emerge from the petal tube, residual pollen is directly presented to pollinators (primary pollen presentation).

**Discussion:**

In *Berchemia*, the petals enclosing the stamens, effectively shield the pollen from extreme environmental conditions. The petal-stamen complex slow movement (first centrifugal, later centripetal) and pollen presentation in *Berchemia* suggest a unique reproductive strategy. This mechanism promotes outcrossing, minimizing interference between the pistil and stamens, and offers reproductive assurance by delayed self-pollination.

## 1 Introduction

The diversity of angiosperm is largely reflected in the diversity of flora structures. Flowers are unique reproductive organs of angiosperms and show higher variability than those of all other groups (Zhang, 2004), making them ideal structures for pollination, fertilisation, and seed setting. Flowers achieve reproductive success through pollination (the pistil receives pollen to produce seeds) and pollen dispersal (the anther of the stamen disperses pollen). Under selection pressure to increase pollination and pollen distribution efficiency, the morphology and structure of stamens and pistils adapt to different pollination patterns, improving both pollination and reproductive success (Barrett, 2002; Ren, 2008), increasing the reproductive capacity of angiosperms (Bynum and Smith, 2001; He et al., 2005; Clark and Husband, 2007; Wang et al., 2010; Ren and Tang, 2012; Wiemer et al., 2012; Song et al., 2013; Abdusalam and Tan, 2014; Li et al., 2022).

Angiosperms can control the timing of pollen presentation and limit the amount of pollen carried by pollinators mainly through packaging and dispensing mechanisms (Harder and Wilson, 1994; Castellanos et al., 2006). Secondary pollen presentation (2PP) is one of the dispensing mechanism. While most plants present pollen directly by their anthers, plants with 2PP present pollen on other floral structures (other than the anthers) or via particular expulsion mechanisms (Howell et al., 1993; Yeo, 1993; Ladd, 1994). This phenomenon has been confirmed in at least 29 families of angiosperms (El Ottra et al., 2024). Pollen transfer typically occurs during the advanced bud stage, after pollen has been released at the advanced bud stage but before stigma reception (i.e., protandry) (El Ottra et al., 2024). The gradual release of pollen via 2PP allows pollinators to remove pollen in batches and deposit it on the stigma prolonging the duration of the male stage and improving the efficiency and accuracy of pollen transfer or deposition onto the stigma. Therefore, 2PP in angiosperms is biologically important for improving the fitness of males and females avoiding interference between male and female functions and promoting heterogametic fertilisation (El Ottra et al., 2024).

Petals are the most characteristic and eye-catching organs in eudicot flowers. The colour, morphology, size, structure, and function of petals have shown a wide variety throughout evolution (Endress and Matthews, 2006; Thien et al., 2009). Although petal diversity has been thought to be related to the different visual attractiveness functions of pollinators (Endress, 1994; Irish, 2009; Whitney et al., 2009a; Irish, 2010; Sulborska et al., 2012; De Craene, 2018; Konarska and Masierowska, 2020), this alone cannot fully explain all floral diversity (Endress, 1994). Beyond attraction, petals protect developing stamens and pistils (Delph, 1996), store nectar (Kramer and Hodges, 2010), apply pollen to pollinators (Yeo, 1993; De Almeida et al., 2013), emit olfactory attractants (Balao et al., 2011), and act as a landing foothold or platform for pollinators (Whitney et al., 2009b; Alcorn et al., 2012; Giovanetti and Aronne, 2013; Katsuhara et al., 2017). Additionally, “corolla dragging,” where withered corolla drag anthers across the stigma, can facilitate delayed selfing (Sun et al., 2005; Qu et al., 2007; Duan et al., 2010).

**Figure 1:**
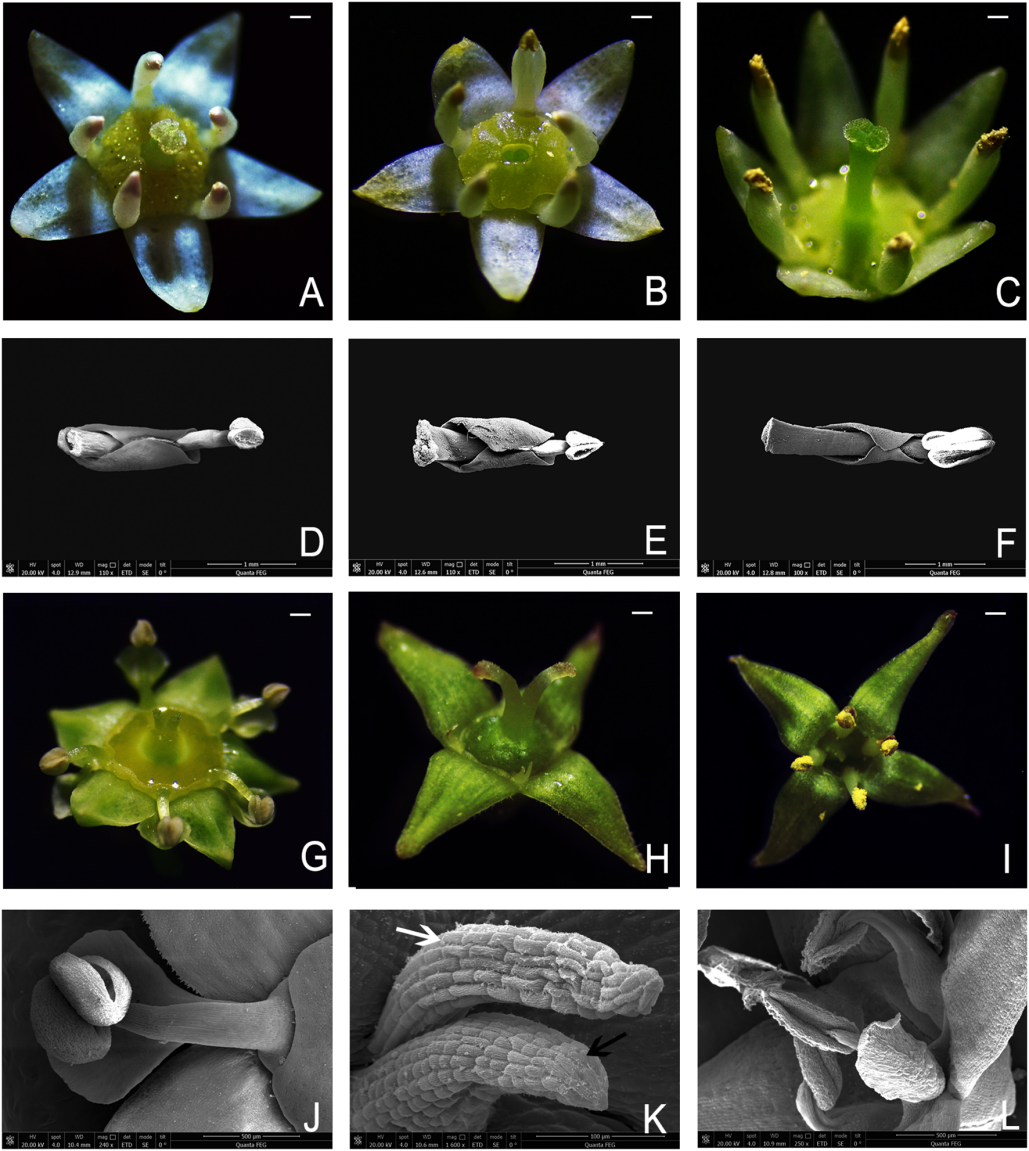
Flowers and petal–stamen complex of several plants in Rhamnaceae. **(A)** Hermaphroditic flower of *B. flavescens*. **(B)** Male flower of *B. flavescens*. **(C)** Hermaphroditic flower of *B. polyphylla* var. *leioclada*. **(D)** Petal–stamen complex of *B. flavescens* (hermaphroditic flower). **(E)** Petal–stamen complex of *B. flavescens* (male flower). **(F)** Petal–stamen complex of *B. polyphylla* var. *leioclada*. (hermaphroditic flower). **(G)** Hermaphroditic flower of *Z. jujuba* var. *spinosa.* **(H)** Female flower of *R. tangutica*. **(I)** Male flower of *R. tangutica*. **(J)** Petal and stamen of *Z*. *jujuba* var. *spinosa.* **(K)** Vestigial petal (white arrow) and stamen (black arrow) in the female flower of *R*. *tangutica*. **(L)** Stamens and petals in the male flower of *R*. *tangutica*. Scale bars = 1 mm.

In most angiosperms, petals and stamens alternate (Gu and Hultgård, 2001). However, in families, such as Vitaceae (Chen et al., 2007; Ma et al., 2021; Parmar et al., 2021; Rabarijaona et al., 2023), Loasaceae (Weigend et al., 2010; Henning and Weigend, 2013; Leite et al., 2016; Acuna et al., 2018; Henning et al., 2018), Berberidaceae (Ying et al., 2011; Li et al., 2022; Zhang et al., 2023), and Rhamnaceae (Medan and D’ambrogio, 1998; Medan, 2003; Calvillo-Canadell and Cevallos-Ferriz, 2007; Chen and Schirarend, 2007; Wang G. et al., 2021; Patel et al., 2024), petals are mostly opposite to the stamens. Moreover, in Rhamnaceae the presence, absence, and morphology of petals vary among the different genera (Chen and Schirarend, 2007). The petals of *Rhamnus* are vestigial. In *Ziziphus*, the petals are obovate or spatulate but do not enclose the stamens. In contrast, in other genera, petals are typically obovate, hooded, or spatulate, and often enclose the stamens to varying degrees (Chen and Schirarend, 2007; **Figure 1**).

In *Berchemia*, the petals opposite the stamens are typically spoon or pocket shaped and curl inwards to enclose the stamens forming a petal–stamen complex. These inward-curled petals lose their ability to attract pollinators. Medan and D’ambrogio (1998) suggested that in andromonoecious *Trevoa quinquenervia*, cucullate petals protect the anthers during the male phase, contributing to pollen delivered in different doses and reducing pollen theft. However, the function of the petal–stamen complex in *Berchemia*, which exhibits both androdioecious and hermaphroditic traits, remains unclear.

In this study, we investigated *B. flavescens* (at least morphologically androdioecious) and *B. polyphylla* var. *leioclada* (hermaphroditic), analysing their floral characteristics, and conducting experiments such as manual petal removal and separation. Our aim was to understand how the components of these plants coordinate to ensure successful pollination process and improve male reproductive fitness. We propose the following hypotheses: (1) *B. flavescens* and *B. polyphylla* var. *leioclada* exhibit 2PP, with petals playing a key role; (2) the slow movement of the petal–stamen complex may be related to the function of “petal dragging”; and (3) inward-curled petals protect the stamens from environmental stress, enhance pollen viability, increase pollen output, and reduce the likelihood of pollen theft.

## 2 Materials and methods

### 2.1 Study site and species

This study primarily utilised live specimens collected from natural and artificial populations. Specimens of *B. flavescens* and *Rhamnus tangutica* were collected from natural populations in the Qinling Mountains, Shaanxi, China (E108°47′32.84″, N33°49′47.53″; 1,803-m elevation), while *Berchemia sinica* and *Hovenia acerba* were collected from another natural population in the Qinling Mountains, Shaanxi, China (E107°45′33.37″, N34°03′38.76″; 1,219-m elevation). Specimens of *B. polyphylla* var. *leioclada* were obtained from artificially cultivated populations at Northwest University, Xi’an, Shaanxi, China (E108°55′39.72″, N34°14′58.61″). Additionally, *Ziziphus jujuba* var. *spinosa* was collected from Qingliangshan Forest Park, Xi’an, Shaanxi, China (E108°55′29.54″, N34°10′29.90″).

The populations of *B. flavescens* and *B. sinica* consist of both hermaphroditic (**Figure 1A**) and male (**Figure 1B**) adult individuals. However, only hermaphroditic adult individuals were found in the populations of *B. polyphylla* var. *leioclada* (**Figure 1C**), *Z. jujuba* var. *spinosa* (**Figure 1G**), and *H. acerba*. In contrast, the population of *R. tangutica* consisted of female (**Figure 1H**) and male (**Figure 1I**) adult individuals.

Voucher specimens of all plants collected for this study were deposited in the Northwest University Herbarium (Xi’an, China) under accession numbers NWU19349 to NWU19353.

### 2.2 Floral syndrome observation

Flowers of *B. flavescens* and *B. polyphylla* var. *leioclada* are yellow-green and actinomorphic, and their five petals are spatulate, obovate, or hood shaped. The five stamens, which are antepetalous, are often enclosed by the petals forming a petal–stamen complex (**Figures 1A–F**). Since the petals of hermaphroditic flowers resemble those of male flowers, we focused on hermaphroditic flowers of *B. flavescens* and *B. polyphylla* var. *leioclada* to observe their floral characteristics. Flowering process, petal morphology features, and stamen changes were observed on 30 tagged well-developed flower buds. In addition, we also observed the morphological characteristics of petals and the changes of stamens in *B. sinica*, *H. acerba*, *R. tangutica*, and *Z. jujuba* var. *spinosa*. Dissected floral organs were photographed with a stereomicroscope (Leica EZ4 W, Germany).

### 2.3 Detection of pollination characteristics

#### 2.3.1 Detection of dichogamy

Thirty flower buds of *B. flavescens* and 30 flower buds of *B. polyphylla* var. *leioclada* were randomly selected and tagged in each population. The state of the flowers and anthers was observed and photographed hourly from 7 a.m. on the day before flowering until the flowers withered.

Pollen viability was determined using a modified Alexander staining method (Peterson et al., 2010). Briefly, pollen was placed in Alexander dye for 15–30 min at 37°C. The number of non-aborted (magenta-red) and aborted pollen grains (blue-green) in each field was counted under a microscope (Nikon Eclipse 50i, equipped with a DS-Fil camera, Japan), and pollen viability was calculated.

Stigmatic receptivity was determined using the benzidine–hydrogen peroxide method (Dafni, 1992). Briefly, flowers in different flowering stages were collected during peak flowering, and the stigmas were immersed in a benzidine–hydrogen peroxide solution [4:11:22, benzidine: 1% hydrogen peroxide: 3% water (v/v/v)] for 3 min. Receptivity was indicated by the presence of bubbles and blue stain. Degrees of receptivity were assigned based on the intensity of the reaction: no reaction (−), weak positive (+), strong positive (++), or very strong positive (+++) (Dafni and Maués, 1998).

#### 2.3.2 Statistics of pollen removal

To investigate pollen presentation strategies, pollination efficiency, and whether the petals act as key organs for 2PP, we empirically studied pollen removal as an indicator of reproductive success in *B. flavescens* and *B. polyphylla* var. *leioclada*. Twenty flower buds (undehisced anthers) were selected and fixed directly in FAA (50%) solution for statistical analysis of pollen grain numbers within the anthers. In addition, 20 flowers of each species were randomly selected to calculate the amount of pollen removed between the pollen was extruded through the cleft or gap in the petal tube and before the anther protruded from the petal tube. After the anthers had fully protruded from the petal tube, the anthers from 20 flowers of each species were removed, and the remaining pollen was counted. When the anthers moved to the top of the stigma, the anthers from 20 flowers of each species were removed, and the remaining pollen was counted.

To count pollen, stamens from each flower were hydrolysed in 8 M NaOH for 10 min and then crushed using a dissecting needle. The volume was brought up to 1 ml using 10% KCl solution. Pollen grains were counted using a microscope (Nikon Eclipse 50i equipped with a DS-Fil camera, Japan). This process was repeated five times.

#### 2.3.3 Observations on visitors

During the peak flowering phase, the species and behaviour of visiting insects were observed and recorded continuously from 7:00 to 18:00 every day over 2–3 days of fine weather. Pollinators were identified by observing their effective contact with the anthers and/or stigmas and confirming the presence of pollen on their bodies. In addition, the flower-visiting behaviour was photographed, and specimens were collected for identification.

#### 2.3.4 Morphology and structure of petals

To investigate how petals, as key organs of 2PP, coordinate with stamens to disperse pollen during pollen presentation, we studied the morphology and structure of petals and examined their development.

Flowers and petals of *B. flavescens* and *B. polyphylla* var. *leioclada* were collected in EP tubes and fixed in 50% FAA. The specimens were then dehydrated through an ethanol series (70%, 85%, 95%, and 100%) and subsequently through a series of anhydrous ethanol–isoamyl acetate mixtures [2:1, 1:1, and 1:2 (all v/v)], finishing with 100% isoamyl acetate. After dehydration, the flowers and petals were then dried in a CO_2_ critical point dryer, sputter-coated with gold, examined and photographed using a Quanta FEG 450 scanning electron microscope.

Flowers at different developmental stages were collected and fixed in 2.5% glutaraldehyde in 0.1 mol L^−1^ phosphate buffer (pH 7.0) at 4°C. Semi-thin sections were prepared using routine methods (Liu et al., 2022; Ma et al., 2024), dehydrated using a gradient series of ethanol, and transitioned through ethanol and 1,2-epoxypropane to 1,2-epoxypropane before being embedded in Epon 812 resin. Subsequently, 2-μm sections were cut using a Leica RM2155 microtome with glass knives. The sections were then stained with toluidine blue O and observed under a microscope (Nikon Eclipse 50i equipped with a DS-Fil camera, Japan).

#### 2.3.5 Effects of artificial treatment on stamens

To verify that the petals mediate pollen removal and the function of “petal dragging” of *B. flavescens* and *B. polyphylla* var. *leioclada*, we conducted empirical research to determine whether the removal or separation of petals affected reproductive success.

One hundred well-developed and open flower buds were randomly selected and labelled, then divided into five treatment groups as follows: (1) petals removed, (2) petals and stamens separated, (3) stigmas destroyed, (4) artificial cross-pollination, and (5) control (no treatment). The position and morphology of the stamens were recorded every hour until the flowers wilted. In addition, the time at which the stamens moved slowly above the stigma after treatment was recorded.

#### 2.3.6 Data analysis

All statistical analyses were performed using SPSS software (version 17.0). Analysis of variance was used to determine whether there was a significant difference in the amount of pollen dispersed between 2PP and PPP, with a significance level set at p < 0.05. Results are presented as mean ± SE. All experiments were repeated three times. Additional statistical analyses were performed using GraphPad Prism 8.0.2.263 software.

## 3 Results

### 3.1 Pollination syndrome

#### 3.1.1 Petal–stamen complex movement and pollen presentation

**Figure 2:**
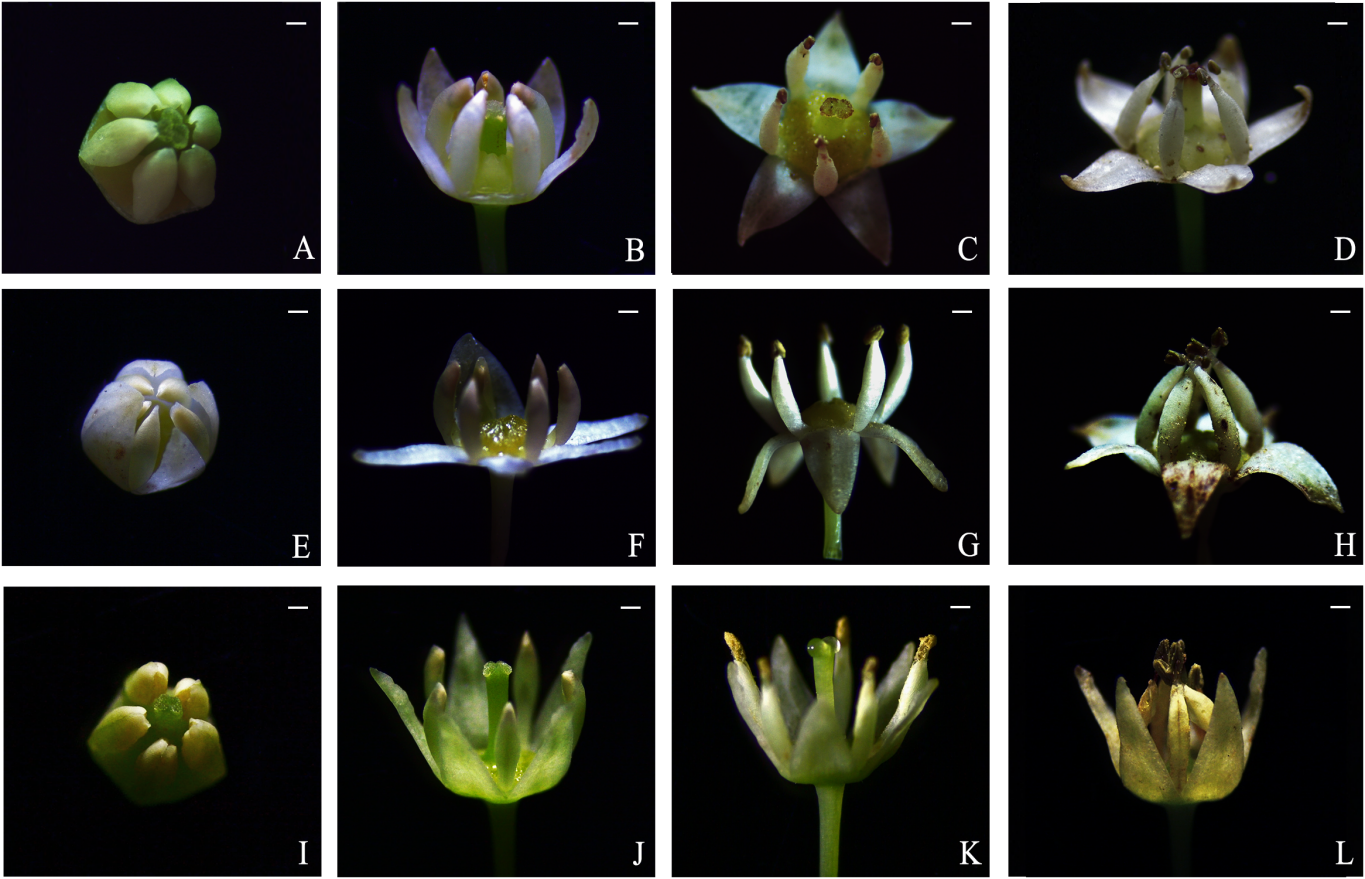
Flower dynamics of *B. flavescens* and *B. polyphylla* var. *leioclada.* **(A–D)** Hermaphroditic flower of *B. flavescens*. **(E–H)** Male flower of *B. flavescens*. **(I–L)** Hermaphroditic flower of *B. polyphylla* var. *leioclada*. Scale bars = 1 mm.

**Figure 3:**
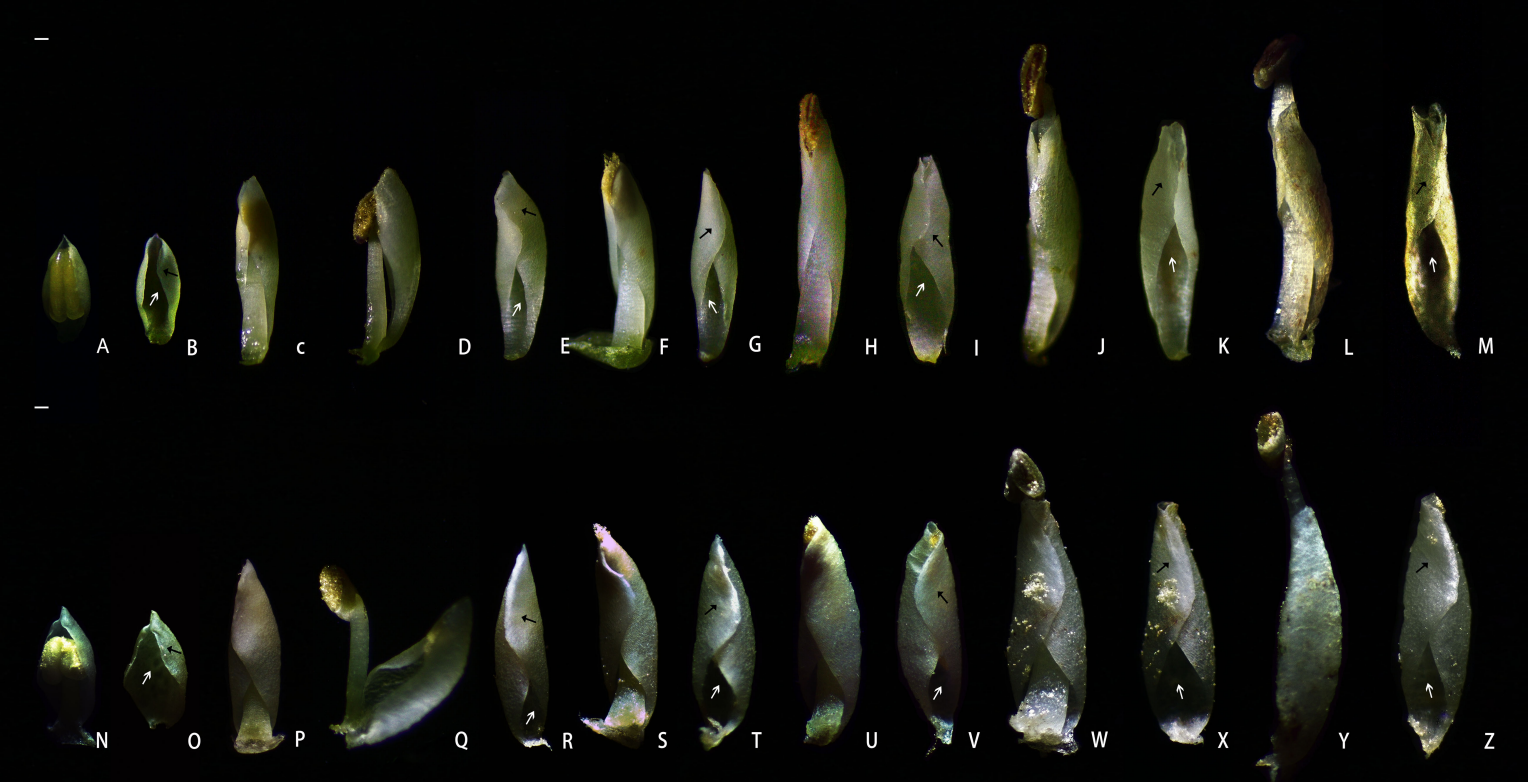
Morphological changes in the petal–stamen complex during pollen presentation of hermaphroditic flowers in *B. polyphylla* var. *leioclada* and *B. flavescens.* **(A–M)** Petal–stamen complex of *B. polyphylla* var. *leioclada* (hermaphroditic flower). **(N–Z)** Petal–stamen complex of *B. flavescens* (hermaphroditic flower). Adaxial surface of the flower petal indicated by a white arrow; abaxial surface of the flower petal indicated by a black arrow. Scale bars = 0.5 mm.

**Figure 4:**
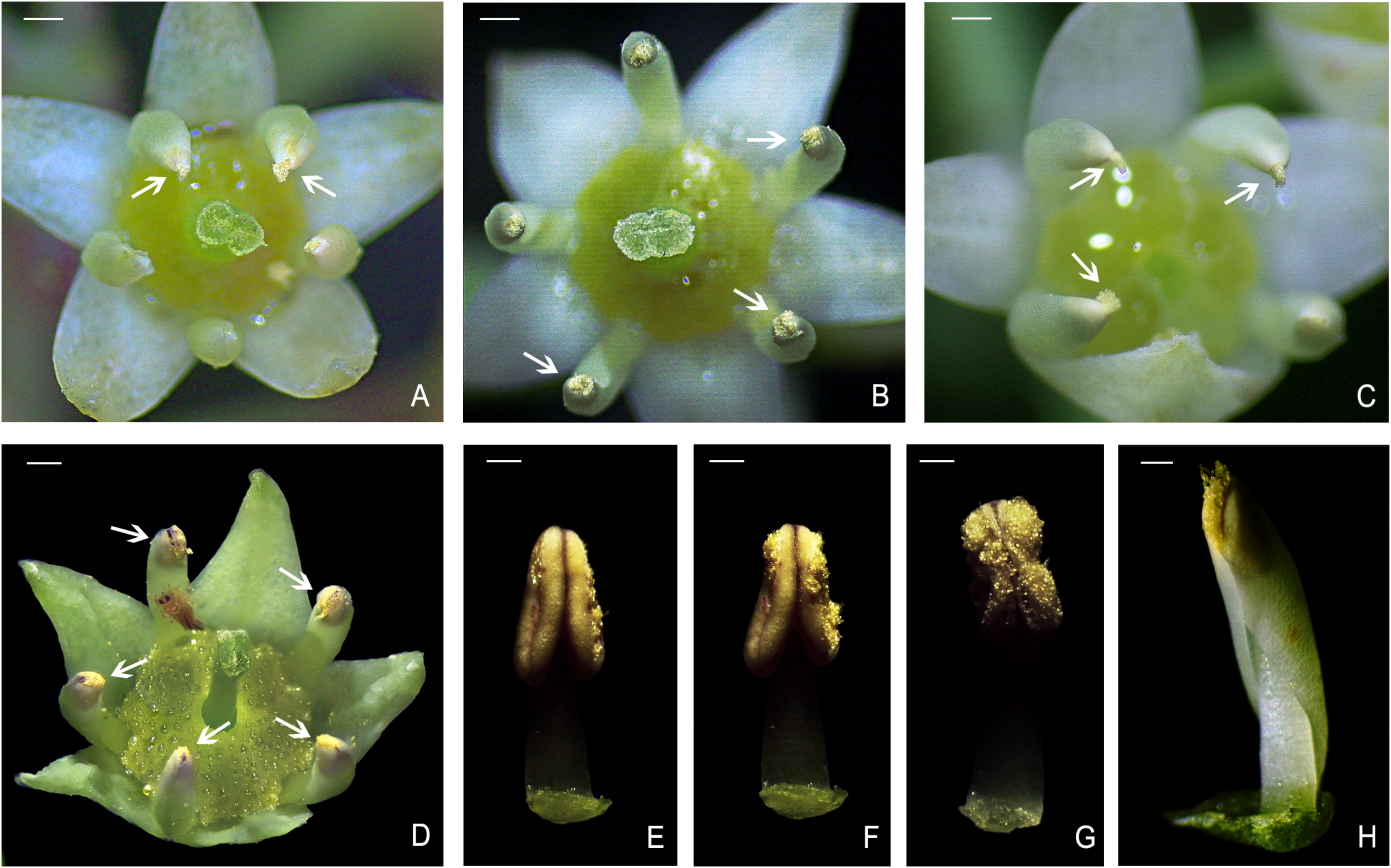
Secondary pollen presentation (2PP) in *B. flavescens* and *B. polyphylla* var. *leioclada*. **(A, B)** Pollens pushed out of the gap in the petal tube of the hermaphroditic flower in *B. flavescens* (arrow). **(C)** Pollens pushed out of the gap in the petal tube of the male flower in *B. flavescens* (arrow). **(D)** Pollens pushed out of the gap in the petal tube of the hermaphroditic flower in *B. polyphylla* var. *leioclada* flower (arrow). **(E–G)** Process of anther dehiscence after petal removal in *B. polyphylla* var. *leioclada.* **(H)** Petal–stamen complex of *B. polyphylla* var. *leioclada.* Scale bars **(A–D)** = 1 mm. Scale bars **(E–H)** = 0.5 mm.

The petals of *B. flavescens* and *B. polyphylla* var. *leioclada* enclosed the stamens forming a petal–stamen complex (**Figures 1A–F**). This complex moved slowly during flowering (**Figure 2**), and the shape of the petals changed constantly (**Figure 3**). During the small flower bud stage, the petals covered or pressed against the anthers and surrounded the pistils (**Figures 3A, B, N, O**). Before blooming, the middle sections of the petals partially overlapped giving them a “cucullate” appearance as they curl inwards (**Figures 3C, P**). At the advanced bud stage, the anthers had already dehisced longitudinally (**Figures 3D, Q**, **4E, F**), releasing pollen grains into the tube formed by the wrapped petals (**Figures 3C, D, P, Q**). As the petals, and filaments continued to elongate, the tops of the cucullate petals appeared as a cleft or gap (**Figures 3F, S**). The filament and cone-shaped anther acted as pistons pushing pollen grains in the petal tube to be extruded out in clumps or clusters through a cleft or gap at the top of the petal tube (2PP; **Figures 4A–D**). As the filaments and petals grew, the anthers gradually protruded from the petal tube, with some pollen remaining in the anthers. The mode of pollen presentation changed from the secondary pollen presentation (2PP) to the primary pollen presentation (PPP). Additionally, the petals gradually transitioned from a “cucullate” to a “shawl-shaped” form (**Figures 3H–M, U–Z**). Initially, the petal–stamen complex moved slowly in a centrifugal direction, which obviously increases the distance between the anther and stigma (**Figures 2A–C, E–G, I–K**). Subsequently, the complex moved slowly in a centripetal direction. Finally, the anthers of hermaphroditic flowers gathered above the stigma (**Figure 2D, L**), and the anthers of male flowers gathered at the centre of the flower (**Figure 2H**).

#### 3.1.2 Pollination characteristics

##### 3.1.2.1 Pollen viability and stigmatic receptivity

The flowers of *B. flavescens* and *B. polyphylla* var. *leioclada* exhibited typical protandrous characteristics. Before blooming (the advanced bud stage), the anthers were enclosed within the petals (anther dehiscence occurred), and pollen grains were first released into the petal tube (**Figures 3C, D, P, Q**). At this point, pollen viability was high, while stigmatic receptivity was almost absent (**Table 1**).

**Table 1:** Pollen viability and stigmatic receptivity of *B. flavescens* and *B. polyphylla* var. *leioclada*.

Species	Items	The sepals were on the verge of unfolding (the advanced bud stage)	Pollen gradually squeezed from the cleft or gap at the top of the cucullate petal tube until just before the anther extended out of the cleft or gap at the top of the cucullate petal tube	The spatial distance between the stamen and the pistil reached its maximum	The petal–stamen complex moves above the stigma
*B. flavescens*	Pollen viability (%)	91.52	90.64	73.30	18.09
	Stigma receptivity	–	++++	+++	+/−
*B. polyphylla var.* leioclada	Pollen viability (%)	95.71	91.29	76.34	21.73
	Stigma receptivity	–	++++	+++	+/−

“+++” indicates stigmas with higher receptivity; “++++” indicates stigmas with the highest receptivity; “+/−” indicates some stigmas have receptivity while others do not; “−” indicates almost absent stigmatic receptivity.

Stigmatic receptivity reached its peak when pollen was extruded from the cleft or gap of the petal tube during the secondary pollen presentation (2PP) stage. As the flowering process advanced, pollen viability gradually declined, and stigmatic receptivity weakened after the anthers emerged from the petal tube cleft. Notably, when the anthers approached the stigma later in the flowering process, a small number of pollen grains remained in the anthers. These remaining pollen grains retained viability, and the stigma maintained some receptivity (**Table 1**).

##### 3.1.2.2 Pollination efficiency and pollen removal

When the anthers of *B. flavescens* and *B. polyphylla* var. *leioclada* dehisced, the released pollen was enveloped by the petal tube (**Figures 3C, D, P, Q**). The pollen presentation process can be divided into two stages. In the first stage (2PP), a cleft or gap created at the top of the petal tube due to the elongation of filaments and petals (**Figures 3F, S**), allowing clusters of pollen grains to be extruded from this opening and gradually presented to pollinators (**Figure 4**). Petals are key organs for 2PP. The time taken for pollen to be pushed out of the petal tube by the anthers until the anther begins to extend the petal tube of *B. flavescens* was approximately 7.643 ± 0.508 h (indicating that 2PP lasts about this duration). During this time, approximately 14,081 ± 253.3 pollen grains were released constituting approximately 73.4% of the total pollen. For *B. polyphylla* var. *leioclada*, the time from when pollen was extruded from the petal tube gap to the point when the anthers began to protrude out of the petal tube was approximately 2.686 ± 0.108 h (indicating that 2PP lasts about this duration). Approximately 7,637 ± 163.6 pollen grains were released accounting for roughly 29.8% of the total pollen.

With the elongation of the filaments and petals, the anthers gradually protruded from the petal tubes (**Figures 3H, J, L, U, W, Y**). Pollens were still present in the anthers at this time, and the mode of pollen presentation changed from the secondary pollen presentation (2PP) to the primary pollen presentation (PPP). The number of pollen grains in *B. flavescens* remaining in the anthers after they protruded from the top of the petal tube was approximately 5,094 ± 141.5. It took approximately 63.5 ± 1.080 h from the beginning of the anther protruding from the petal tube to slow the movement above the stigma. In *B. polyphylla* var. *leioclada*, approximately 17,947 ± 467.4 pollen grains remained in the anthers when the anther protruded from the petal tube; the duration is approximately 58.514 ± 4.022 h until they slowly move above the stigma.

##### 3.1.2.3 Nectary disc and floral visitors

**Figure 5:**
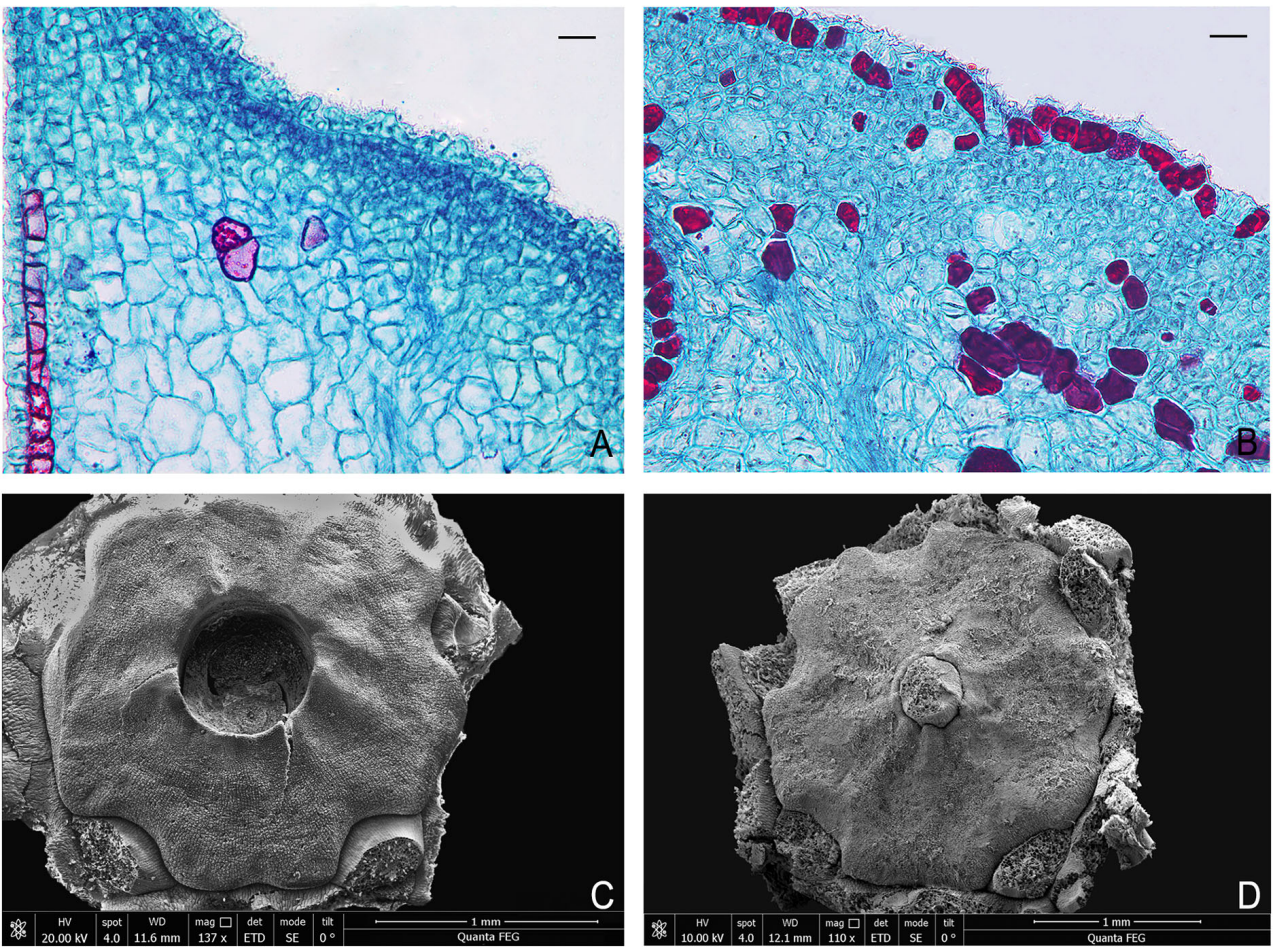
Disc and disc nectary of *B. flavescens* and *B. polyphylla* var. *leioclada.* **(A)** Longitudinal section of the disc of hermaphroditic flower in *B. flavescens*. **(B)** Longitudinal section of the disc of hermaphroditic flower in *B. polyphylla* var. *leioclada*. **(C)** SEM of the disc in *B. flavescens* (hermaphroditic flower). **(D)** SEM of the disc in *B. polyphylla* var. *leioclada* (hermaphroditic flower). Scale bars = 50 μm.

**Figure 6:**
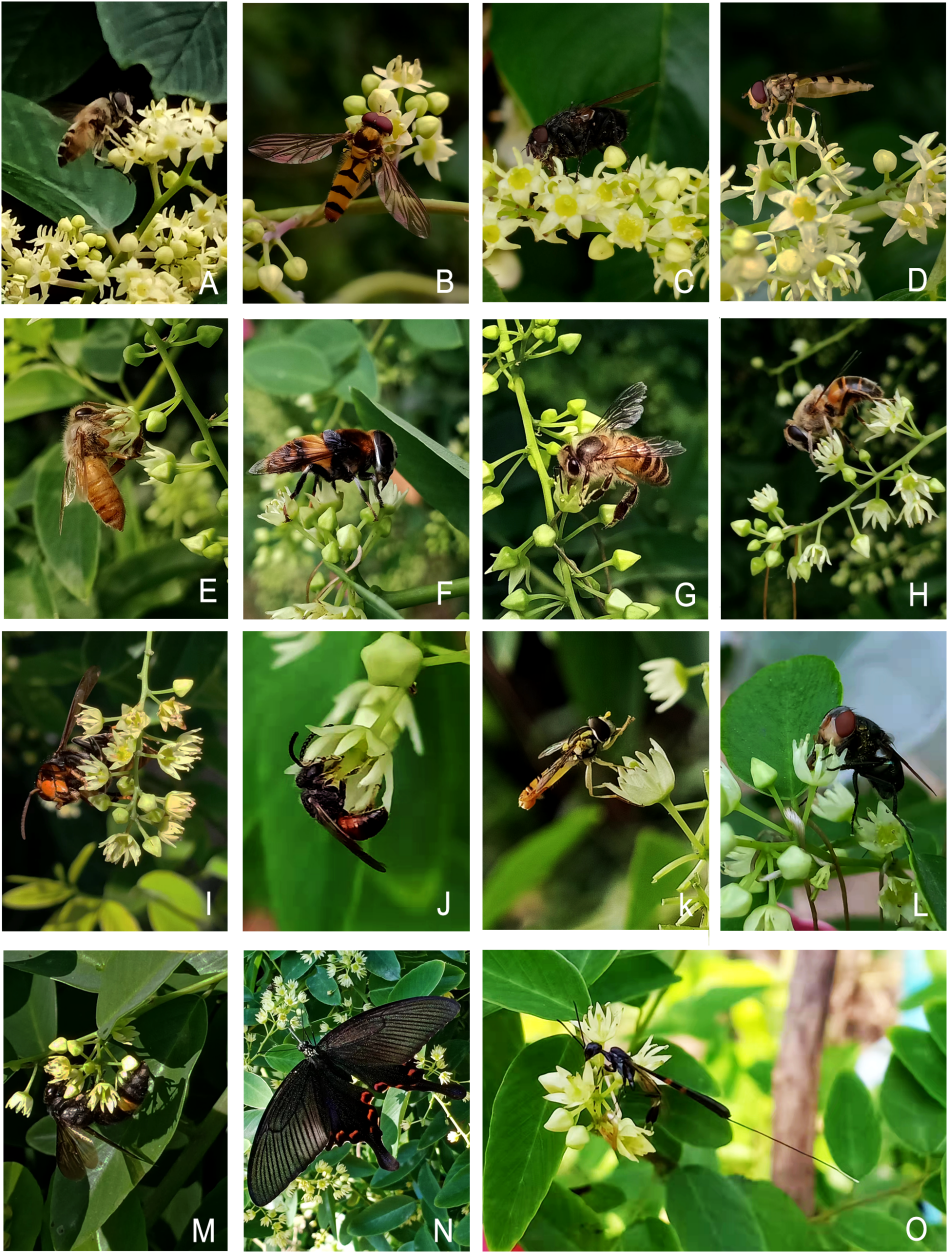
Flower-visiting insects of *B. flavescens* and *B. polyphylla* var. *leioclada*. **(A)** Apidae. **(B)** Syrphidae. **(C)** Muscidae. **(D)** Syrphidae. **(E)** Apidae. **(F)** Syrphidae. **(G, H)** Apidae. **(I)** Vespidae. **(J)** Crabronidae. **(K)** Syrphidae. **(L)** Calliphoridae. **(M)** Scoliidae. **(N)** Papilionidae. **(O)** Gasteruptiidae.

The floral nectaries around the base of the ovary in *B. flavescens* and *B. polyphylla* var. *leioclada* belong to disc nectary (**Figure 5**). The nectary is usually composed of a secretory epidermis, nectariferous tissue, and vascular bundles. The epidermal cells are nearly square or rectangular and arranged neatly. The nectariferous tissue is composed of three to six layers of small, polygonal cells with large nuclei and dense cytoplasm (**Figures 5A, B**). After flowering, colourless and transparent nectar is visible on the surface of the flower disc, attracting floral insects (**Figures 4A–D**). In the population of *B. flavescens*, approximately 19 insect species from seven orders were recorded (**Figures 6A–D**). The largest order was Diptera, which accounted for 36.9% of the total insect visitors, followed by Hymenoptera (26.3%), Coleoptera (15.8%), and Lepidoptera, Hemiptera, Araneae, and Acarina accounted for 5.25% each. Approximately 31 insect species were observed in *B. polyphylla* var. *leioclada* belonging to five orders (**Figures 6E–O**). The largest order was Hymenoptera accounting for 35.5% of the total insect visitors, followed by Diptera (32.3%), Lepidoptera (22.6%), Hemiptera (6.4%), and Neuroptera (3.2%).

##### 3.1.2.4 Micromorphology and structure of mature petals

**Figure 7:**
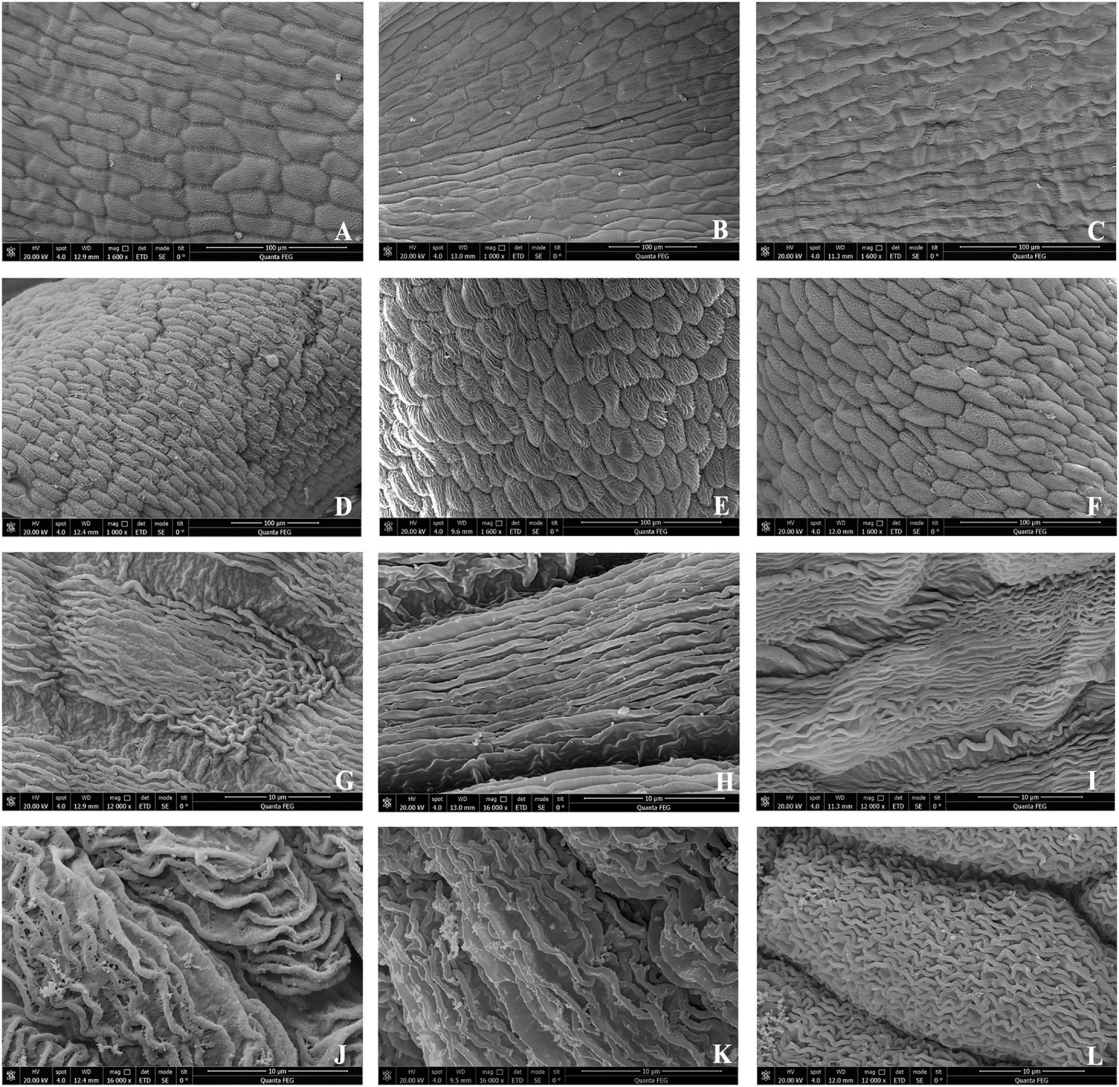
SEM showing the surface of the petals in *B. flavescens* and *B. polyphylla* var. *leioclada*. **(A, G)** Adaxial surface of the male flower petal of *B. flavescens*. **(D, J)** Abaxial surface of the male flower petal of *B. flavescens*. **(B, H)** Adaxial surface of the hermaphroditic flower petal of *B. flavescens*. **(E, K)** Abaxial surface of the hermaphroditic flower petal of *B. flavescens*. **(C, I)** Adaxial surface of the hermaphroditic flower petal of *B. polyphylla* var. *leioclada*. **(F, L)** Abaxial surface of the hermaphroditic flower petal of *B. polyphylla* var. *leioclada*.

The mature petal epidermal cells of *B. flavescens* (both male and hermaphroditic) and *B. polyphylla* var. *leioclada* (hermaphroditic) predominantly displayed an arrangement of flat rectangles, spindles, or fusiform shapes. The adaxial epidermal cells exhibited numerous longitudinal, straight, and fine stripes, with a few displaying wavy stripes (**Figures 7A–C, G–I**). However, the abaxial epidermal cells primarily featured transverse, oblique, or twisted stripe arrangements (**Figures 7D–F, J–L**).

**Figure 8:**
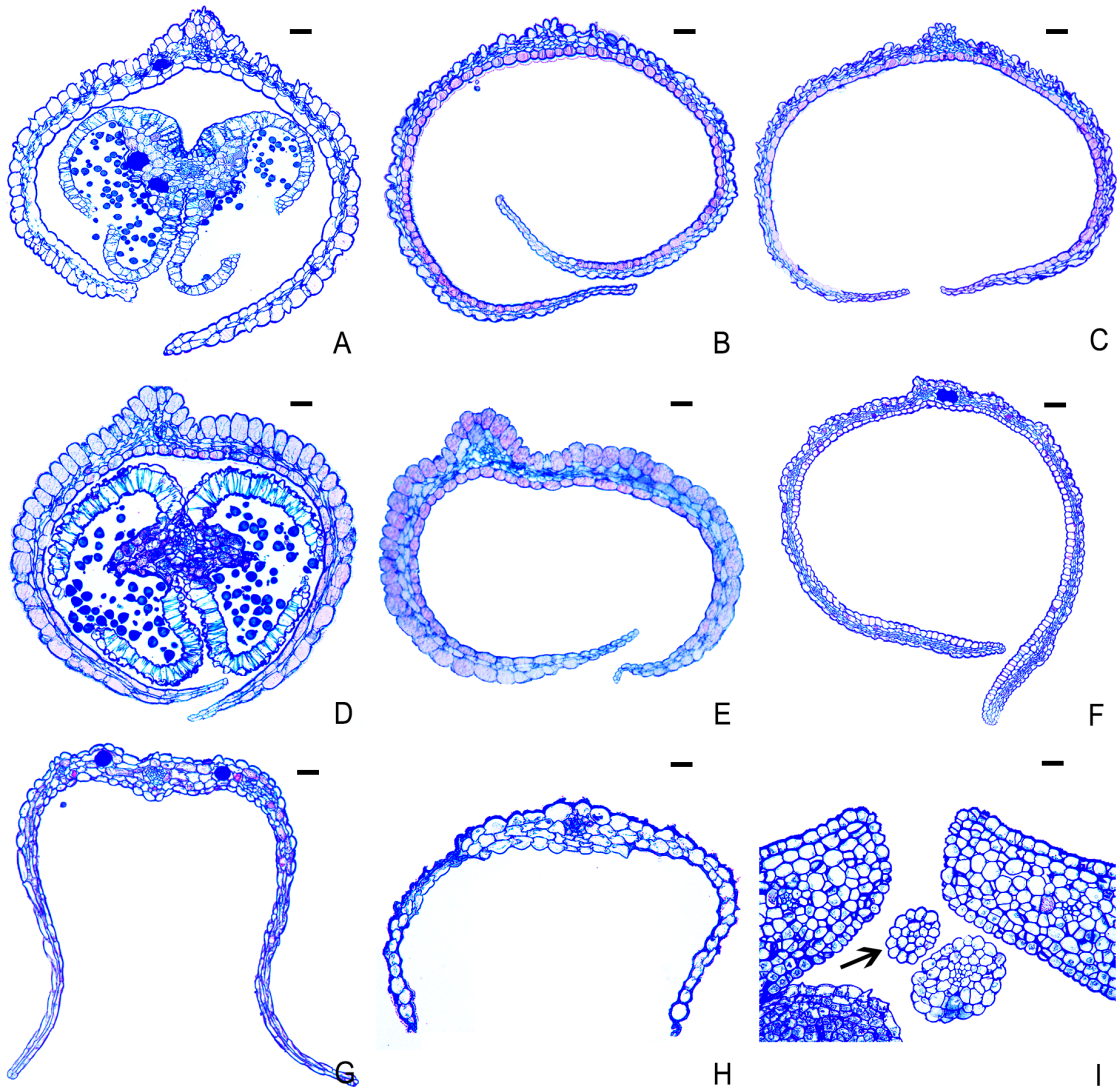
Transverse sections of petals of several plants in Rhamnaceae. **(A)** *B. polyphylla* var. *leioclada* (hermaphroditic flower). **(B)** *B. flavescens* (hermaphroditic flower). **(C)** *B. flavescens* (male flower). **(D)** *B. sinica* (male flower). **(E)** *B. sinica* (hermaphroditic flower). **(F)** *H. acerba* (hermaphroditic flower). **(G)** *Z. jujuba* var. *spinosa* (hermaphroditic flower). **(H)** *R. tangutica* (male flower). **(I)** *R. tangutica* (female flower, arrow indicates petal). Scale bars = 10 μm.

Transverse sections of mature petals revealed that the petals of *B. flavescens* (male and hermaphroditic flowers), *B. sinica* (male and hermaphroditic flowers), *B. polyphylla* var. *leioclada* (hermaphroditic flowers), *H. acerba* (hermaphroditic flowers), and *Z. jujuba* var. *spinosa* (hermaphroditic flowers) were composed of two layers of epidermal cells (upper and lower), parenchyma cells, and vascular tissues. The epidermal cells were larger in volume and had thicker cytoplasm, whereas the parenchyma cells of the mesophyll were smaller and exhibited an irregular shape. The volume of epidermal cells in the middle area of the petal was notably larger than that at the edge of petal (**Figures 8A–G**). In the middle sections of petals, approximately three to five layers of parenchyma cells were observed between the upper and lower epidermis, whereas the edge regions contained either no parenchyma cells or a single layer (**Figures 8A–G**). In contrast, the petals of *R. tangutica* were vestigial (**Figures 8H, I**). Specifically, the transverse sections of female petals were oval in shape (**Figures 8I**).

Additionally, the adaxial surface of the petals appeared relatively smooth and flat, whereas the abaxial surface was characterised by numerous bumps (**Figures 7**, **8**).

##### 3.1.2.5 Effect of artificial treatment on petal–stamen complex movement

**Figure 9:**
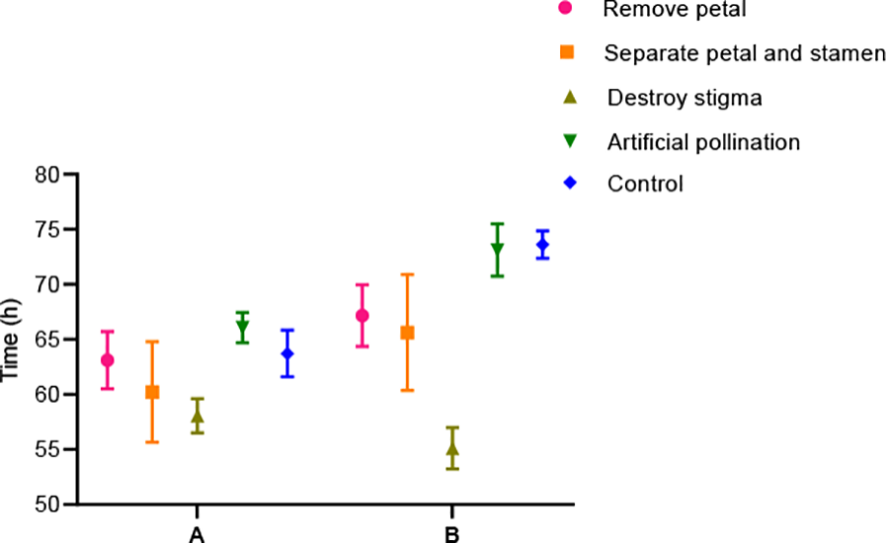
Time for stamen movement to reach above/near the stigma in the hermaphroditic flowers of *B. flavescens* and *B. polyphylla* var. *leioclada* after artificial treatments. **(A)** *B. polyphylla* var. *leioclada*. **(B)** *B. flavescens*.

**Figure 10:**
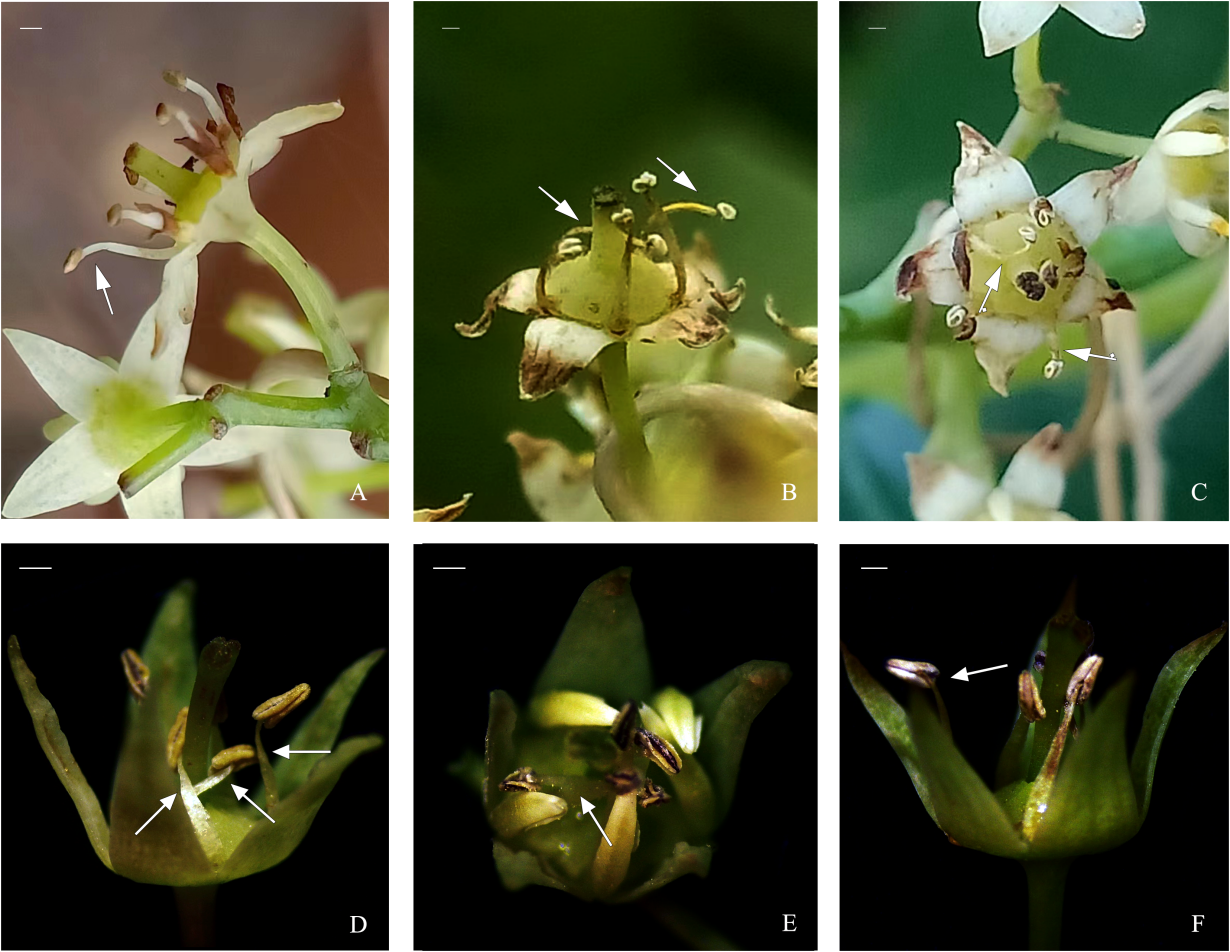
Effects of artificial petal removal and separation of the petal–stamen complex on stamen movement in hermaphroditic flowers of *B. flavescens* and *B. polyphylla* var. *leioclada*. **(A–C)** *B. flavescens*. **(D–F)** *B. polyphylla* var. *leioclada*. Arrows indicate those stamens that curl outwards and lie flat or recline on the flower disc. Scale bars = 1 mm.

After conducting treatments—petal removal, separation of petals and stamens, destruction of stigmas, artificial cross-pollination, and a control group (no treatment), we observed significant changes in the movement of the petal–stamen complex relative to the stigma (**Figure 9**). Compared with the control and artificial cross-pollination treatments, the time for stamens to return to the stigma was the shortest in the stigma treatment. In addition, when the petals were removed or the petals and stamens were separated, the stamens of some *B. flavescens* and *B. polyphylla* var. *leioclada* failed to gather near the stigma. In these cases, the stamens curl outwards and lie flat or recline on the flower disc (**Figure 10**).

## 4 Discussion

### 4.1 Unique pollen presentation strategies in *Berchemia*

The male fitness of angiosperms largely depends on the “fate” of their pollen, with successful plant reproduction relying on the efficient transfer of a significant amount of pollen between plants. However, in species with granular pollen, less than 1% of the pollen removed from the anther successfully reaches the stigma (Stanton et al., 1992; Harder, 2000). This low male fitness has driven plants to evolve strategies that enhance pollination accuracy leading to the development of diverse pollen presentation mechanisms.

In *B. flavescens* and *B. polyphylla* var. *leioclada*, pollen presentation can be divided into two stages: 2PP and PPP. The cone-shaped anthers, with a pointed upper end and wide lower end, provide space for pollen release (**Figures 3C, P**, **4E–H**). Pollen grains were first released into the petal tube after anther dehiscence. The cone-shaped anther and filament acted as a piston and gradually presented the pollen in the petal tube to pollinators by pushing out the pollen in clumps or clusters from below to above, rather than releasing it simultaneously. This mode manifested as 2PP mechanism. Petals play a crucial role in 2PP process. The petals on the adaxial surface are relatively smooth and flat (**Figures 7**, **8**), which reduces the friction between the pollen and petals and is conducive to the extrusion of pollen from the petal tube. As the anthers gradually protrude from the petal tube, the remaining pollen is directly presented to pollinators by the anthers, manifested as PPP mechanism. At the end of flowering, the petal–stamen complex moves centripetally, positioning above the stigma, allowing the unpollinated pistil the opportunity for selfing.

*Berchemia* employs a complex pollen presentation strategy that combines 2PP and PPP, a phenomenon not yet reported in other plant taxa (Fan et al., 2015; Yang et al., 2019; El Ottra et al., 2024). The evolution of the petals in *Berchemia* is adapted to this mode of pollination.

### 4.2 Effects of petal–stamen complex movement on mating patterns

Owing to the sessile growth habit of flowering plants, sexual reproduction depends on successful pollen transfer. From this point of view, the fate of the pollen (that is, whether the pollen can be transferred to the stigma of the pistil) determines the fate of the future flower. Plants have evolved elaborate flowers to promote outcrossing and increase the genetic diversity of their offspring. Although plants bloom in large numbers to attract pollinators, this inevitably leads to selfing. Breeding strategies of outcrossing and selfing not only exist in different plants but also in many species with mixed mating systems that delay selfing. The decisive factors influencing mating modes include the distance between anthers and stigmas, spatial changes in stamens or pistils, and temporary flower closures (petal movement) (Dole, 1992; Barrett, 2003). Many plants achieve pollination and secure reproduction by the movement of their floral organs.

In *B. flavescens* and *B. polyphylla* var. *leioclada*, the petal–stamen complex exhibits slow movements during flowering. Anthers have dehisced while the complex surrounds the pistil at the advanced bud stage (**Figures 3C, D, P, Q**). However, as the petals curl inwards on both sides and enclose the stamens, pollen grains are released into the petal tube to prevent selfing. As the flower gradually opens and the petals and filaments grow, pollen is slowly squeezed out of the cleft at the top of the cucullate petal tube. During this period, the petal-stamen complex moves in the centrifugal direction, and the distance between pistil and stamen increases significantly (**Figures 2A–C, E–G, I–K**). This movement effectively hinders selfing. Subsequently, the petal–stamen complex of hermaphroditic flowers moves back in the centripetal direction to a position above the stigma (**Figures 2D, L**) allowing the unpollinated pistil the opportunity for selfing. This mechanism, where selfing through anther–stigma contact occurs after the loss of outcrossing opportunities, exemplifies “delayed selfing” (Ippolito and Armstrong, 1993; Sun et al., 2005; Duan et al., 2007; Qu et al., 2007). In this study, pollen was gradually presented from the beginning of pollen release to the end of the petal–stamen complex movement in *B. flavescens* and *B. polyphylla* var. *leioclada* extending the duration of pollen release and enhancing male reproductive fitness through pollen gradual presentation. According to the reproductive assurance hypothesis, the fitness advantage of self-pollination is that cross-pollination occurs first when outcrossing opportunities arise; However, when cross-pollination is not feasible, self-pollination occurs (Aarssen, 2000). Therefore, *B. flavescens* and *B. polyphylla* var. *leioclada* exhibit mixed mating systems where delayed selfing serves as a reproductive assurance strategy in environments where cross-pollination is limited. This floral design and the behaviour of the petal–stamen complex promote outcrossing, avoiding interference between pistils and stamens, and enable delayed selfing, ensuring reproduction (Ren and Tang, 2012; Armbruster et al., 2014; Goodwillie and Weber, 2018; Liu et al., 2020; Abdusalam et al., 2021; Minnaar and Anderson, 2021; Silva-Batista et al., 2021).

### 4.3 Functional evolution of petals in Rhamnaceae

Petal diversity is widely believed to play a role in attracting pollinators (Whitney et al., 2009a; Sulborska et al., 2012; De Craene, 2018; Konarska and Masierowska, 2020). However, this function alone cannot fully explain the extensive floral diversity observed across species (Endress, 1994). In *Berchemia*, the petals are positioned opposite the stamens, enclosing them to provide protection and enhance pollen output efficiency, albeit at the cost of reduced ability to attract pollinators. The presence of open disc nectaries, which are less selective for nectar-feeding insects, suggests that *B. flavescens* and *B. polyphylla* var. *leioclada* attract a diverse range of flower-visiting insects. Consequently, these species experience high pollinator diversity and abundance (**Figures 5**, **6**).

Pollen is released through the petal tube ensuring that each pollinator collects only a small amount of pollen per visit. This mechanism minimizes pollen waste, prolongs the pollen release period, and enhances pollination accuracy (Thomson et al., 2000). Therefore, the secondary pollen presentation (2PP) mechanism, resulting from the stamens being enclosed by the petals, represents an effective adaptive strategy in *Berchemia* for optimising reproductive success in the context of diverse and abundant pollinator interactions.

In this study, mature petals of *B. flavescens* and *B. polyphylla* var. *leioclada* exhibited differences in cell growth rates between the middle and edge regions. Cells in the middle region of the petal grew faster than the cells at the edge resulting in a significant expansion force in the middle of the petals, while the edges of the petals are subjected to a tensile force. In addition, the number of adaxial epidermal cells in the petal was lower than that of the abaxial epidermal cells, which is conducive to the sides of the petals curling inwards. The petals gradually curled inwards towards both sides and eventually enclosed the stamens under the involution force caused by this uneven growth. After removing the petals or separating the petals and stamens, some of the stamens curl outwards and lie flat or recline on the flower disc indicating that the anthers did not gather above the stigma (**Figure 10**). This phenomenon indirectly verified the “dragging effect” of the petals indicating that the dragging of petals enhances selfing accuracy in these two species. The dragging effect of petals promotes the slow movement of the petal–stamen complex, thereby promoting cross-pollination, avoiding the interference between the pistils and stamens, and achieving delayed selfing, thereby ensuring reproductive success for the plants.

Pollen is highly susceptible to damage from UV-B radiation and high temperatures caused by direct sunlight (Feng et al., 2000; Sato et al., 2002; Aylor, 2004). For plants without pollen protection measures, the direct exposure of pollen to sunlight (especially strong UV-B radiation) can result in varying degrees of pollen damage (Zhang et al., 2014). Flat epidermal cells have weaker light absorption abilities than conical epidermal cells (Noda et al., 1994; Gorton and Vogelmann, 1996). In *B. flavescens* and *B. polyphylla* var. *leioclada*, flat petal epidermal cells were observed instead of conical ones (**Figure 7**), illustrating the protective effect of petals on pollen. The petals opposite the stamens, allowing the petals to enclose the stamens as they curl inwards, effectively shielding them from rainwater erosion and intense UV-B radiation, likely prolonging pollen vitality, and improving the pollen output rate.

In Rhamnaceae, petals are mostly opposite the stamens, but their presence or absence and shapes vary among the different genera (Chen and Schirarend, 2007; **Figure 1**). Moreover, the ability of petals to attract pollinators is greatly degraded in Rhamnaceae. However, the evolution of petals is closely related to the way the pollen is presented and the type of breeding system. In *Berchemia*, which exhibits androdioecious (at least morphologically androdioecious) and hermaphroditic breeding system, the 2PP formed by petals enclosing the stamens effectively prolongs the time of pollen presentation, reduces interference between stamens and pistils through petal dragging, and promotes cross-pollination and delayed selfing. In *Ziziphus*, which has hermaphroditic flowers, petals do not enclose stamens but, instead, droop outwardly in late flowering stages (**Figure 1G**). This mechanism is not intended to promote delayed selfing; rather, it aims to prevent self-pollination and enhance outcrossing rates (Wang F. et al., 2021). This suggests a typical PPP mechanism in *Ziziphus*, contrasting with those of plants in *Berchemia*, showing the significant role of petals in the flowering process. Moreover, in *Rhamnus* (dioecy), the petals are vestigial forming rod-like shapes or even disappear (Chen and Schirarend, 2007). This may be the result of adaptation to complete outcrossing.

Therefore, the unique pollen presentation strategy caused by the inwards curl of the petals of *Berchemia* may represent a derived floral trait. In Rhamnaceae, the pollen presentation strategy may have evolved from the simplest form—primary pollen presentation (where petals do not enclose the stamens) to a complex pollen presentation strategy combining primary and secondary pollen presentation (with petals enclosing the stamens) and then to complete outcrossing (petals vestigial). This evolution may lead to outcrossing and delayed selfing, characterised by vestigial petal.

## Data Availability

The raw data supporting the conclusions of this article will be made available by the authors, without undue reservation.
